# Identification of a Rat Mammary Tumor Risk Locus That Is Syntenic with the Commonly Amplified 8q12.1 and 8q22.1 Regions in Human Breast Cancer Patients

**DOI:** 10.1534/g3.118.200873

**Published:** 2019-03-26

**Authors:** Cody Plasterer, Shirng-Wern Tsaih, Angela Lemke, Rebecca Schilling, Melinda Dwinell, Andrea Rau, Paul Auer, Hallgeir Rui, Michael J. Flister

**Affiliations:** * Genomic Sciences and Precision Medicine Center, Medical College of Wisconsin, Milwaukee, WI; † Department of Physiology, Medical College of Wisconsin, Milwaukee, WI; ** Department of Pathology, Medical College of Wisconsin, Milwaukee, WI; ‡ Joseph J. Zilber School of Public Health, University of Wisconsin-Milwaukee, Milwaukee; § GABI, INRA, AgroParisTech, Université Paris-Saclay, 78350, Jouy-en-Josas, France

**Keywords:** breast cancer, risk, incidence, outcome, TCGA

## Abstract

Breast cancer risk is 31% heritable, yet the majority of the underlying risk factors remain poorly defined. Here, we used F2-linkage analysis in a rat mammary tumor model to identify a novel 11.2 Mb modifier locus of tumor incidence and burden on rat chromosome 5 (chr5: 15.4 – 26.6 Mb). Genomic and RNA sequencing analysis identified four differentially expressed candidates: *TMEM68*, *IMPAD1*, *SDCBP*, and *RBM12B*. Analysis of the human syntenic candidate region revealed that *SDCBP* is in close proximity to a previously reported genetic risk locus for human breast cancer. Moreover, analysis of the candidate genes in The Cancer Genome Atlas (TCGA) revealed that they fall within the commonly amplified 8q12.1 and 8q22.1 regions in human breast cancer patients and are correlated with worse overall survival. Collectively, this study presents novel evidence suggesting that *TMEM68*, *IMPAD1*, *SDCBP*, and *RBM12B* are potential modifiers of human breast cancer risk and outcome.

Breast cancer affects 1 in 8 women worldwide, resulting in 600,000 deaths annually (Jemal *et al.* 2011). In most cases, a single cause of breast cancer cannot be found, but rather multiple environmental and genetic factors contribute to overall disease susceptibility and outcome. This, combined with complex gene interaction in both malignant tumor cells and nonmalignant tumor microenvironment cells, poses significant challenges in identifying new modifiers of breast cancer risk and outcome.

One strategy to identify heritable modifiers of breast cancer is through chromosome substitution (*i.e.*, consomics). This approach was recently used to characterize mammary tumor risk by transferring chromosomes from the tumor-resistant BN rat onto the tumor susceptible SS rat background (Adamovic *et al.* 2010). Following a single carcinogenic exposure, >90% of SS rats developed mammary tumors, whereas the BN rats developed no macroscopically detectable mammary tumors in the same timeframe. Substitution of the BN-derived rat chromosome 5 (RNO5) into the SS background (*i.e.*, SS-5BN consomic) significantly lowered mammary tumor incidence to 50% (*P* < 0.05) (Adamovic *et al.* 2010). Notably, this study did not resolve location of the genetic modifier(s) beyond the chromosomal level and the factor(s) on RNO5 that alter mammary tumor risk remain unknown.

In this study, we used F2 linkage analysis of SS and SS-5BN consomic rats to localize an 11.2 Mb (chr5: 15.4 – 26.6 Mb) quantitative trait locus (QTL) for mammary tumor incidence and burden. Importantly, this region overlaps with a human breast cancer risk locus (Kuchenbaecker *et al.* 2014) and is syntenic with the commonly amplified 8q12.1 and 8q22.1 regions in human breast cancer patients in The Cancer Genome Atlas (TCGA). Genomic and RNA sequencing analysis identified four candidate genes within the RNO5 QTL: *TMEM68*, *IMPAD1*, *SDCBP*, and *RBM12B*. In TCGA breast cancer patients, all four genes were significantly associated with worse overall survival and expression was predominantly attributed to copy number alterations (CNA). Collectively, these data suggest that one or more of the candidate genes likely contribute to the risk and outcome of human breast cancer patients.

## Materials and Methods

### F2 Linkage Analysis of Mammary Tumor Incidence and Burden

The Institutional Animal Care and Use Committee of the Medical College of Wisconsin approved all procedures involving live animals. The SS/JrHsdMcwi (SS) rat strain was crossed with the SS-5BN consomic strain to yield F1 offspring, which were then intercrossed to yield 64 (SS x SS-5BN)F2 (F2) female rats. Mammary tumors were induced by a single oral gavage of 7,12-dimethylbenz[a]anthracene (DMBA) in sesame seed oil (65mg/kg) that was administered to the F2 female rats between 49 to 55 days-of-age. Rats were palpated every week to record tumor incidence and latency. At 15 weeks, rats were killed and tumors were collected, weighed, and snap-frozen for further analysis. A total of 25 simple sequence length polymorphism (SSLP) markers were used for chromosome-wide genotyping of rat chromosome 5 (RNO5). Marker distances were calculated using a high-resolution rat genetic map (Littrell *et al.* 2018). A single-locus QTL scan was performed and LOD scores were calculated at 0.5 cM interval across rat chromosome 5, using the imputation method implemented in R/qtl (Sen and Churchill 2001) and significance was determined on the basis of 1000 permutations of the data (Churchill and Doerge 1994). A LOD score exceeding the 0.1 chromosome-wide adjusted threshold was considered significant (Lander and Kruglyak 1995). The Bayes credible interval function in R/qtl (bayesint) was used to approximate the 95% confidence intervals for the QTL peak location for both the additive and the interactive models, as described in (Solberg *et al.* 2004).

### RNAseq Analysis

Total RNA was extracted by Trizol from whole tumors that were excised from SS and SS-5BN consomic rats (n = 6 per group), followed by library preparation using Illumina’s TruSeq RNA library kit and sequencing on an Illumina HiSeq2500 (Illumina, Inc., San Diego, CA). The Trim Galore program (v0.4.1) was used to trim bases with a Phred quality score <20 and only reads with a Phred quality score equal or higher than 20 were taken for analysis. The RSEM program function “rsem-prepare-reference” (v1.3.0) was used to extract the transcript sequences from the rat genome (build Rnor6.0) (Li and Dewey 2011) and to generate Bowtie2 indices (Bowtie2 v2.2.8) (Langmead and Salzberg 2012), followed by read alignment using the “rsem-calculate-expression” function. Differential expression analysis was performed using the Bioconductor package DESeq2 version 1.12.4 (Love *et al.* 2014) to compute log2 fold changes and false discovery rate-adjusted p-values. Statistical significance was determined at a false discovery rate (FDR) threshold of 0.05.

### Rat Genomic Sequencing Analysis

Genomic sequence of the BN/NHsdMcwi and SS/JrHsdMcwi rat strains was accessed from the Rat Genome Database (http://rgd.mcw.edu/) and has been described in detail elsewhere (Flister *et al.* 2014).

### Comparative Genomics Analysis

The human genomic regions that are syntenic with the RNO5 QTL (chr5: 15.4 – 26.6 Mb) were identified using the Virtual Comparative Map (VCMap) tool available at the Rat Genome Database (http://vcmap.animalgenome.org/). The genomic, epigenomic, and transcriptomic features of the syntenic human regions (8q12.1 and 8q22.1) were then examined in the breast cancer cohort in The Cancer Genome Atlas (TCGA-BRCA) using the EDGE in TCGA tool (Rau *et al.* 2017) and cBioPortal (Gao *et al.* 2013; Cerami *et al.* 2012). RNAseq data from 888 female breast cancer patients from the TCGA-BRCA cohort and the corresponding clinical parameter data were downloaded from the Broad GDAC Firehose (https://gdac.broadinstitute.org/) using the R package TCGA2STAT (http://www.liuzlab.org/TCGA2STAT/). The maximally selected rank statistics from the ’maxstat’ R package (https://www.r-project.org/) was used to determine the optimal cutpoint for dichotomization (high *vs.* low) of expression values of the candidate genes (*i.e.*, *TMEM68*, *IMPAD1*, *SDCBP*, and *RBM12B*). The prognostic value of the resulting dichotomized mRNA expression was evaluated using the Log-rank test and Kaplan-Meier curves. A Cox proportional hazards model was used to evaluate the prognostic value of dichotomized mRNA expression with outcome.

### Statistical analysis

All statistical analyses were performed as described above in the R statistical environment (version 3.5.0).

### Data Availability

File S1 contains detailed descriptions of all supplemental files. File S2 contains variant annotations and locations on rat chromosome 5 (RNO5). File S3 contains the RNAseq gene expression data. The raw gene expression data are available at SRA with the accession number: PRJNA504606. Supplemental material available at Figshare: https://doi.org/10.25387/g3.7323950.

## Results and Discussion

### Interval mapping of a mammary tumor QTL on RNO5

**Figure 1:**
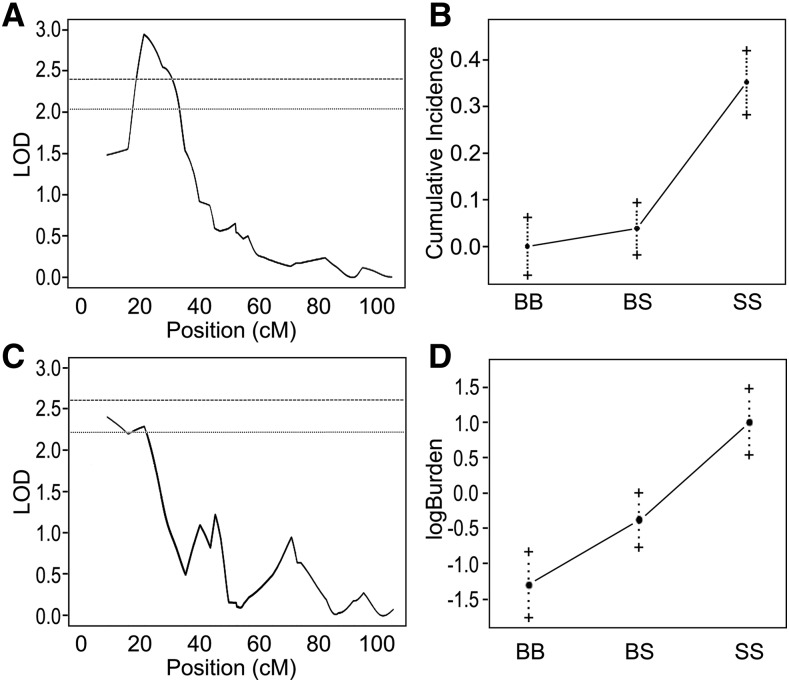
Interval mapping of mammary tumor incidence and burden in F2 progeny from a cross between SS and SS-5BN rats. Genotypes were determined at 25 polymorphic markers for 64 phenotypically defined F2 females. (A) Distribution of LOD scores for tumor incidence at 6 weeks post-exposure to DMBA identified a candidate region between 16.9 cM - 34.4 cM on RNO5. (B) A logistic regression with the peak marker in the candidate region revealed a significant association with tumor incidence at 6 weeks post-exposure to DMBA for the SS parental strain. (C) Distribution of LOD scores for tumor burden at 15 weeks post-exposure to DMBA identified an overlapping candidate region between 8.9 cM and 27.9 cM on RNO5. (D) A logistic regression with the peak marker in the candidate region revealed a significant association with tumor burden at 15 weeks post-exposure to DMBA. Lines indicate permutation-derived thresholds for significance at *P* = 0.05 (hashed lines) and *P* = 0.1 (dotted line).

To begin localizing the genetic modifier(s) of mammary tumor risk on RNO5 (Adamovic *et al.* 2010), phenotype-genotype relationships were assessed using 64 young female (SS x SS-5BN consomic) F2 rats that were administered a single oral dose of DMBA carcinogen (65 mg/kg) between 49 to 55 days-of-age. Interval mapping (IM) with 25 SSLP markers was then used to assess mammary tumor incidence, burden, and latency. At 6 weeks post-exposure to DMBA, a locus for mammary tumor incidence was detected at 21.5 cM (confidence interval: 16.9 - 34.4 cM; LOD = 2.9; *P* < 0.05) (Figure 1A), with the SS allele at the peak marker (D5Rat124) being significantly correlated with the highest mammary tumor incidence (*P* < 0.05) (Figure 1B). Likewise, an overlapping locus associated with mammary tumor burden was detected at 8.9 cM (confidence interval: 8.9 - 27.9 cM; LOD = 2.4; *P* < 0.1) at 15 weeks post-exposure to DMBA (Figure 1C), with the SS allele at the peak marker (D5Rat187) being significantly correlated with the greatest tumor burden (*P* < 0.05) (Figure 1D). No locus for mammary tumor latency when considering later time points up to 15 weeks post-exposure to DMBA was detected on RNO5. Collectively, these data suggest that modifier(s) of early tumor development and growth, leading to increased tumor burden, likely reside within the overlapping interval from 16.9 cM to 27.9 cM, which corresponds to 15.4 Mb to 26.6 Mb on RNO5, respectively.

### Prioritization of candidate genes by genomic and RNA sequencing

The genomic sequences of the SS/JrHsdMcwi (SS) and BN/NHsdMcwi (BN) parental rat strains were also examined within the candidate region (chr5: 15.4 - 26.6 Mb). This revealed a total of 15,722 single nucleotide polymorphisms (SNPs) between the strains, of which 115 SNPs were coding and 17 SNPs in 11 conserved genes were predicted to cause nonsynonymous changes (File S2). None of the 11 conserved genes with nonsynonomous SNPs were predicted to alter protein function, suggesting that the causative allele(s) within the RNO5 QTL (15.4 - 26.6 Mb) are likely noncoding and potentially function through altered gene expression.

**Figure 2:**
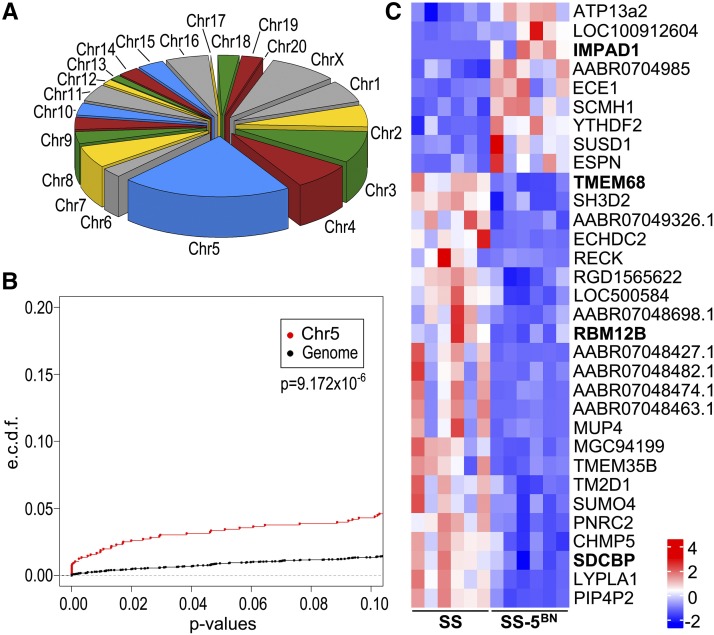
RNAseq analysis of mammary tumors from SS and SS-5BN consomic rats (n = 6 per group). (A) Chromosomal distribution of the 155 differentially expressed genes in mammary tumors isolated fromSS and SS-5BN consomic rats. (B) Distributions of adjusted p-values for differentially expressed genes on RNO5 *vs.* the rest of the rat genome were tested by a two-sample Kolmogorov–Smirnov test. (C) Heatmap of differentially expressed genes that are localized to RNO5. Genes falling within the candidate region are labeled in bold.

Gene expression in SS and SS-5BN consomic tumors (n = 6 per group) was assessed by RNAseq to begin prioritizing potentially causative alleles within the candidate region. A total of 155 differentially expressed (DE) genes were identified in SS-5BN tumors compared with SS tumors (File S3). Overall, DE genes were significantly enriched on RNO5 (32 genes) compared to the genome-wide average (*P* < 0.05; Figure 2A), as were the distributions of p values for RNO5 *vs.* the rest of the genome (*P* = 9.172 × 10e-6; Figure 2B). Of the 32 DE genes that reside on RNO5, four were localized to the 11.2 Mb (chr5: 15.4 – 26.6 Mb) candidate region (Figure 2C). These data imply that heritable genetic alleles on RNO5 are the predominant drivers of DE genes and that the four DE genes residing within the candidate region are potentially causative.

Several important considerations should be made when interpreting the abovementioned genomic and transcriptomic analyses of the QTL region. First, nonsynonymous SNPs may effect protein function in ways that are not yet understood and caution should be exercised before completely ruling out these candidates prior to QTL fine-mapping or functional analysis. Second, it is important to note that RNAseq expression data from already developed tumors might not reflect the genes that are active and expressed in the normal tissue at the time of tumor initiation. Rather, gene expression in the already formed tumors might be more indicative of modified tumor growth and progression. Thus, future studies will also be required to assess the temporal expression of the causative candidate(s) that are localized by fine-mapping of the QTL. Finally, there are several highly conserved regions within the QTL that are poorly annotated or consist of gene deserts, which ultimately will also require fine-mapping studies to determine whether these regions contribute to the phenotypes associated with the RNO5 QTL.

### Comparative analysis of syntenic regions within the human genome

The 11.2 Mb (chr5: 15.4 – 26.6 Mb) region contains 45 conserved genes and is syntenic to two regions in the human genome, the 8q12.1 region (chr8:54.6 - 61.7 Mb) and the 8q22.1 region (chr8:93.7 - 96.1 Mb). The human 8q12.1 region was previously associated with risk of HER2+ breast cancer in BRCA2 carriers (Kuchenbaecker *et al.* 2014), whereas no genetic associations of 8q22.1 region with breast cancer risk have been reported. For the 8q12.1 region, the thymocyte specific transcription factor, *TOX*, was reported as the gene closest to the tagged risk allele, rs4305889 [G] (Kuchenbaecker *et al.* 2014). However, *TOX* is not highly expressed in normal or malignant breast epithelium in the Human Protein Atlas (Uhlen *et al.* 2015) and was not among the list of DE genes within the syntenic 11.2 Mb (chr5: 15.4 – 26.6 Mb) region in the rat. In comparison, the neighboring candidate gene, *SDCBP*, is highly expressed in breast cancer cells and was significantly downregulated in SS-5BN tumors compared to SS tumors. Moreover, *SDCBP* and *TOX* fall within the same topologically associated domain (TAD), suggesting that the intergenic SNP, rs4305889 [G], might alter breast cancer risk through altered expression of another proximal candidate, such as *SDCBP*.

In addition to early tumor incidence, the 11.2 Mb RNO5 candidate region was also associated with mammary tumor burden (*i.e.*, aggressiveness), prompting us to explore whether the top candidates within the region were associated with outcome in TCGA-BRCA patients. Surprisingly, expression of all four DE candidates were significantly associated with breast cancer outcome: *TMEM68* (HR = 1.50, *P* = 0.044), *IMPAD1* (HR = 2.10, *P* = 0.003), *SDCBP* (HR = 1.61, *P* = 0.004), and *RBM12B* (HR = 1.46, *P* = 0.022) (Table 1). Using EDGE (Rau *et al.* 2017), we explored the potential drivers for candidate gene expression variance among TCGA-BRCA patients. This revealed that the predominant source of gene expression variance in TCGA-BRCA patients was copy-number alterations (CNA): *TMEM68* (60% of variance), *IMPAD1* (64% of variance), *SDCBP* (24% of variance), and *RBM12B* (30% of variance) (Table 1). TCGA-BRCA data also revealed that both syntenic regions are commonly amplified in human breast cancer patients: 8q12.1 (8% of cases) and 8q22.1 (15% of cases). Collectively, these data suggest that *TMEM68*, *IMPAD1*, *SDCBP*, and *RBM12B* are also potentially modifiers of breast cancer aggressiveness.

**Table 1:** Analysis of outcome associated with candidates in TCGA-BRCA and the main drivers of expression

				Outcome		Proportion of Explained Variance in Gene Expression					
Gene	Chr	Strand	TSS	HR	p-value	CNA	miRNA	TF	Methyl	Genetic	Residual
*TMEM68*	8	—	56651302	1.50	0.044	0.600	0.019	0.096	0.004	0	0.282
*IMPAD1*	8	—	57870487	2.10	0.003	0.645	0.007	0.099	0	0	0.250
*SDCBP*	8	+	59465727	1.61	0.004	0.242	0.014	0.034	0.008	0	0.702
*RBM12B*	8	—	94743730	1.46	0.022	0.303	0.030	0.054	0.006	0	0.607

Chr, Chromosome; TSS, Transcription Start Site; HR, Hazard Ratio; CNA, Copy Number Alteration; TF, Transcription Factor.

Taken together, the data presented in this study provide novel evidence that *TMEM68*, *IMPAD1*, *SDCBP*, and *RBM12B* are potential modifiers of breast cancer incidence and outcome. *SDCBP* (also known as Syntenin) is a PDZ domain scaffolding protein that binds Syndecan (Grootjans *et al.* 1997) and regulates exosome formation (Baietti *et al.* 2012). *SDCBP* has been associated with worse breast cancer outcome that has been attributed to multiple mechanisms, including tumor cell proliferation, invasiveness, and evasion of the antitumor immunity (Liu *et al.* 2018; Yang *et al.* 2013; Qian *et al.* 2013; Koo *et al.* 2002). To our knowledge, this study is the first to link *SDCBP* with a potential role in breast cancer incidence, which is supported by its close proximity to the rs4305889 [G] risk allele (Kuchenbaecker *et al.* 2014). Compared with *SDCBP*, very little is known about the potential role(s) of *TMEM68*, *IMPAD1*, and *RBM12B* in breast cancer or any other relevant disease, with a total of 11 publications existing for these genes in the PubMed database (https://www.ncbi.nlm.nih.gov/pubmed). Nonetheless, the independent evidence in human and rat that implicate *TMEM68*, *IMPAD1*, and *RBM12B* in breast cancer risk and incidence warrant future studies to determine whether they are mechanistically linked to breast cancer or were simply “guilty by association” due to close proximity or co-amplification with *SDCBP*.
